# Leucine Zipper-bearing Kinase promotes axon growth in mammalian central nervous system neurons

**DOI:** 10.1038/srep31482

**Published:** 2016-08-11

**Authors:** Meifan Chen, Cédric G. Geoffroy, Hetty N. Wong, Oliver Tress, Mallorie T. Nguyen, Lawrence B. Holzman, Yishi Jin, Binhai Zheng

**Affiliations:** 1 Department of Neurosciences, School of Medicine, University of California San Diego, La Jolla, California, 92093, USA; 2 Renal Electrolyte and Hypertension Division, University of Pennsylvania, Perelman School of Medicine, Philadelphia, Pennsylvania 19104, USA; 3 Section of Neurobiology, Division of Biological Sciences, and Howard Hughes Medical Institute, University of California San Diego, La Jolla, California, 92093, USA

## Abstract

Leucine Zipper-bearing Kinase (LZK/MAP3K13) is a member of the mixed lineage kinase family with high sequence identity to Dual Leucine Zipper Kinase (DLK/MAP3K12). While DLK is established as a key regulator of axonal responses to injury, the role of LZK in mammalian neurons is poorly understood. By gain- and loss-of-function analyses in neuronal cultures, we identify LZK as a novel positive regulator of axon growth. LZK signals specifically through MKK4 and JNKs among MAP2Ks and MAPKs respectively in neuronal cells, with JNK activity positively regulating LZK protein levels. Neuronal maturation or activity deprivation activates the LZK-MKK4-JNK pathway. LZK and DLK share commonalities in signaling, regulation, and effects on axon extension. Furthermore, LZK-dependent regulation of DLK protein expression and the lack of additive effects on axon growth upon co-manipulation suggest complex functional interaction and cross-regulation between these two kinases. Together, our data support the possibility for two structurally related MAP3Ks to work in concert to mediate axonal responses to external insult or injury in mammalian CNS neurons.

Originally cloned from the human cerebellum, Leucine Zipper-bearing Kinase (LZK, also known as MAP3K13) is a Mitogen-Activated Protein Kinase Kinase Kinase (MAP3K) that signals through the MAPK cascade known to orchestrate cellular responses to extracellular stimuli^1^. The structural features of double leucine/isoleucine zippers and a catalytic domain that is a hybrid between serine/threonine and tyrosine protein kinases render LZK a member of the Mixed Lineage Kinase (MLK) family of MAP3Ks^1^,^2^. Among the MLKs, LZK is closest to Dual Leucine zipper-bearing Kinase (DLK, also known as MAP3K12), sharing ~90% amino acid sequence identity in the kinase domain and the leucine zipper domain that mediates homodimerization critical for kinase activation^3^.

LZK and DLK are the two vertebrate homologues of DLK-1 in *Caenorhabditis elegans* and Wallenda/DLK in *Drosophila melanogaster*. Invertebrate DLK-1 and Wallenda/DLK are known to play multiple roles in the developing and mature nervous systems, such as synaptic development and growth, regeneration of the proximal axonal segment and Wallerian degeneration of the distal segment following axonal injury^4^,^5^,^6^,^7^,^8^,^9^,^10^. To date, studies in the mammalian nervous system have almost exclusively focused on DLK. In the developing nervous system, murine DLK regulates neuronal migration and axonal projection^11^,^12^,^13^, and promotes axon degeneration and neuronal apoptosis^14^,^15^. Notably, DLK has recently emerged as a major signaling hub under conditions of neuronal insults, where it plays a multitude of roles that are highly dependent on the cellular and environmental context and may even appear paradoxical^16^. In neonates, DLK promotes axotomy-induced facial motoneuron death^17^. In the adult, DLK promotes excitotoxicity-induced hippocampal neuron death^18^, both Wallerian degeneration and axon regeneration of dorsal root ganglion (DRG) neurons after peripheral nerve injury^6^,^19^, and both cell death and axon regeneration of retinal ganglion cells after optic nerve injury^20^,^21^.

In contrast to DLK, the biological function(s) and regulation of LZK remain surprisingly underexplored. The high structural and sequence conservation between LZK and DLK, together with their possible endogenous interaction in the adult mouse brain as detected by mass spectrometry^18^, suggests related biochemical and functional properties between these two proteins in the nervous system. In this context, all previous biochemical characterization of LZK signaling resorted to the use of exogenously expressed substrates in non-neuronal cell lines^1^,^22^,^23^, raising the question on the biological relevance of the role of LZK in neurons. Functionally, only one published study implicated LZK as a possible negative regulator of axon growth downstream of Nogo, a myelin-associated axon growth inhibitor^24^.

In this study, we demonstrate that LZK promotes axon growth in mouse neuroblastoma cells and primary CNS neurons by both gain- and loss-of-function analyses through a combination of transient overexpression, RNA interference (RNAi) and gene deletion. We found that in neuronal cells LZK signals through MKK4 and JNKs as the endogenous downstream MAP2K and MAPK effectors, respectively. Consistent with a positive regulatory role of LZK in axon growth, axonal elongation in primary cerebellar neurons is accompanied by upregulation of the endogenous LZK-MKK4-JNK pathway and requires LZK. Neuronal activity deprivation elicits LZK-dependent activation of JNKs, suggesting LZK as a regulator of axon dynamics in response to neuronal insults. Finally, co-manipulation of LZK and DLK reveal functional interaction and cross-regulation at the protein level implicating coordinate regulation of axonal responses to injury by this pair of kinases.

## Results

### LZK promotes neurite growth in mouse neuroblastoma cells

**Figure 1:**
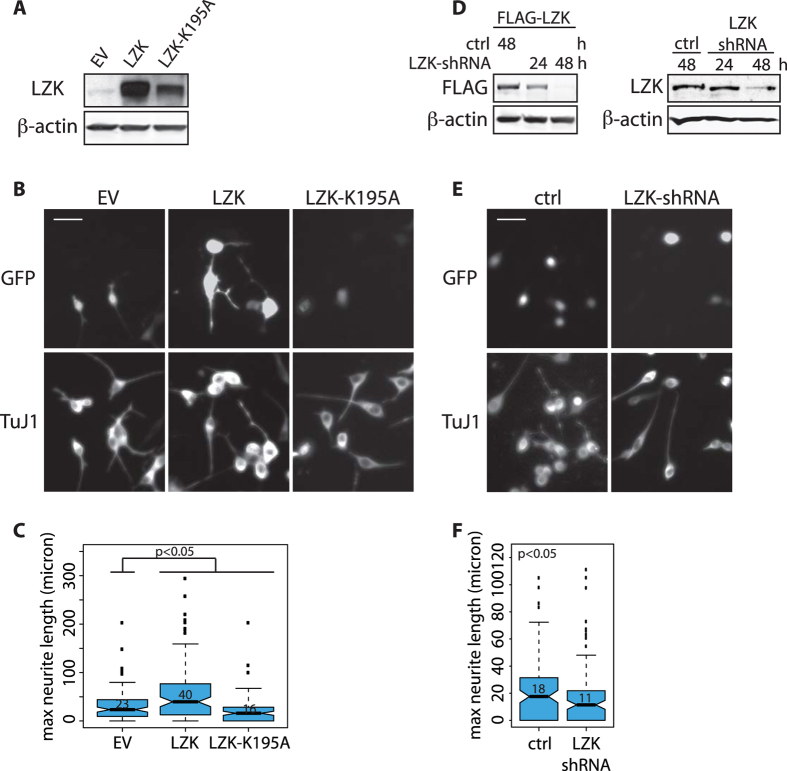
LZK promotes neurite growth in N2a cells in a cell-autonomous manner. (**A**) N2a cells were transiently transfected with GFP-coexpressing pBI empty vector (EV), pBI-LZK, or pBI-LZK-K195A catalytically inactive mutant. Total cell lysates were immunoblotted for LZK to confirm exogenous LZK expression 24 hours after transfection. For all immunoblots presented in this paper, gels shown in the same figure were run under the same experimental conditions. Blots shown within bounded regions were not cropped and spliced together. (**B**) N2a cells transfected with pBI empty vector (EV), pBI-LZK, or pBI-LZK-K195A were immunostained for TuJ1. Immunofluorescence images show GFP labeling of cells expressing the indicated pBI vectors and TuJ1 staining of cells with neuronal identity. Scale bar = 20 μm. (**C**) Graph compares the median maximum (max) neurite lengths of GFP and TuJ1-double positive N2a cells transfected with the indicated pBI vectors. Measurement was based on GFP. Median values are shown for each condition within the graph. Boxplot edges extend to the 25^th^ and 75^th^ percentiles; whiskers extend to non-outliner extremes; points beyond whiskers represent outliners. *p*-values by Wilcoxon test, n > 100 neurons per condition. (**D**) *Left*, Knockdown efficiency of GIPZ-LZK-shRNA coexpressing GFP was tested against exogenous FLAG-LZK in N2a cells. Cells were co-transfected with FLAG-LZK and GIPZ-LZK-shRNA. Total lysates were collected at the indicated times post-transfection and immunoblotted for the indicated proteins. *Right*, To test the knockdown efficiency on endogenous LZK, N2a cells were transfected with GFP-coexpressing GIPZ-nonsilencing-shRNA (ctrl) or pGIPZ-LZK-shRNA. At the indicated times after transfection, total lysates were immunoblotted for endogenous LZK. (**E**) N2a cells transfected with GIPZ-nonsilencing-shRNA (ctrl) or GIPZ-LZK-shRNA were immunostained for TuJ1. Representative immunofluorescence images show GFP labeling of transfected cells and TuJ1 staining of cells with neuronal identity. Scale bar = 20 μm. (**F**) Graph compares the median maximum (max) neurite lengths of GFP- and TuJ1- double positive N2a cells transfected with the indicated vectors. Measurement was based on GFP. Median values are shown for each condition within the graph. Boxplot edges extend to the 25^th^ and 75^th^ percentiles; whiskers extend to non-outliner extremes; points beyond whiskers represent outliners. *p*-values by Wilcoxon test, n > 100 cells per condition.

As a first step to test LZK in cell-intrinsic control of axon growth, we examined the effects of manipulating its expression on neurite extension in mouse neuro-2a (N2a) neuroblastoma cells. For gain-of-function analyses, we expressed mouse wild-type LZK or a catalytically inactive (i.e. kinase dead) mutant LZK-K195A^23^,^24^ using a construct (pBI-CMV3) that coexpresses a variant of green fluorescent protein, ZsGreen (referred to as GFP below for simplicity), both to mark transfected cells and to help track neurite growth (Fig. 1A,B). The longest, or maximum, neurite lengths of cells doubly positive for GFP (indicative of exogenous LZK expression) and neuron-specific class III β-tubulin (TuJ1) were measured based on GFP as described^25^,^26^. Cells overexpressing LZK had nearly a twofold increase in the median maximum neurite length compared to the control, whereas those expressing the catalytically inactive LZK-K195A had significantly shorter neurites – representing a ~30% decrease – than the control (Fig. 1C). These results suggest that LZK-K195A acts as a dominant negative mutant, likely by forming inactive dimers/oligomers with wild-type LZK molecules^23^.

For loss-of-function analyses, we used a construct co-expressing GFP and LZK-specific shRNA that depleted either FLAG-tagged LZK or endogenous LZK with ~80% knockdown efficiency in N2a cells (Fig. 1D). ShRNA-mediated LZK knockdown decreased the median maximum neurite length to ~60% of that observed in the control (Fig. 1E,F). These results indicate that LZK promotes neurite outgrowth in N2a cells and that the catalytically inactive mutant LZK exerts a dominant negative effect that may interfere with endogenous LZK function.

### MKK4 and JNKs are the endogenous downstream effectors of LZK and positively feedback on LZK protein levels in mouse neuroblastoma cells

**Figure 2:**
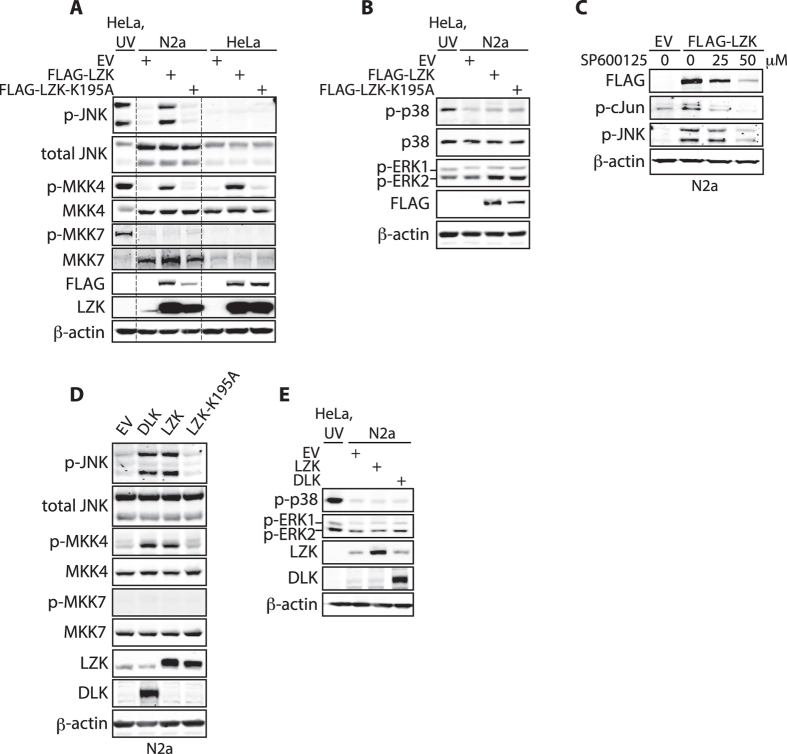
LZK signals through endogenous MKK4-JNKs and JNK inhibition reduces LZK protein levels in N2a cells. (**A**) N2a and HeLa cells were transfected with empty vector (EV), FLAG-LZK, or FLAG-LZK-K195A. HeLa cells collected 45 min after UV irradiation at 45 J/m^2^ served as positive control for activation of endogenous JNK1/2, p38, and MKK4. Total cell lysates were immunoblotted for the indicated proteins. p-JNK indicating phospho-(Thr183/Tyr185)-JNK1/2; p-MKK4 indicating phospho-(Ser257)-MKK4. (**B**) Total lysates from N2a cells transfected with empty vector (EV), FLAG-LZK, or FLAG-LZK-K195A were immunoblotted for the indicated proteins. p-P38 indicates phospho-(Thr180/Tyr182)-p38; p-ERK1/2 indicates phospho-(Thr202/Tyr204)-ERK1/2. (**C**) N2a cells were transfected with empty vector (EV) or FLAG-LZK and treated with SP600125 at the indicated doses at the time of transfection. Total cell lysates were immunoblotted for the indicated proteins. p-cJun indicates phospho-(Ser63)-c-Jun. (**D**) Total lysates of N2a cells overexpressing empty vector (EV), DLK, LZK, or LZK-K195A were immunoblotted for the indicated proteins. (**E**) Total lysates of N2a cells overexpressing empty vector (EV), DLK, or LZK were immunoblotted for the indicated proteins.

Given that LZK promotes neurite extension in N2a cells, we next sought to delineate its endogenous downstream effectors in these cells. Of the three conventional families of MAPKs at the bottom tier of the MAPK cascade (JNKs, p38s and ERKs), wild-type LZK, but not the kinase dead mutant LZK-K195A, induced activating phosphorylation of only endogenous JNKs using antibodies that detect phosphorylated threonine 183 and tyrosine 185 (Fig. 2A,B). Of the two MAP2Ks that are upstream of JNKs (MKK4/SEK1 and MKK7), LZK specifically induced activation of endogenous MKK4, as assayed by its activating phosphorylation at serine 257 (Fig. 2A). These results identify MKK4 and JNKs as the primary downstream effectors of the LZK signaling cascade in N2a cells. Furthermore, LZK-dependent activation of JNKs appears to feedback positively to LZK levels, as JNK inhibition by SP600125^27^ significantly decreased the protein levels of FLAG-LZK (Fig. 2C and S2A). Such an effect on protein levels of exogenous LZK was not observed with pharmacological inhibition of another MAPK member p38 by compound SB203580 (Fig. S2A). Furthermore, at the concentrations of SP600125 used, the activity of p38 was not noticeably altered (Fig. S2B), further supporting the specificity for the effect of JNK activity in maintaining LZK protein levels. Because this plasmid-based expression of LZK is not subjected to endogenous mechanisms of transcriptional or translational regulation, SP600125-dependent reduction in FLAG-LZK protein expression suggests protein destabilization.

A previous study identified both MKK4 and MKK7 as substrates of LZK using overexpressed MAP2Ks in the monkey kidney fibroblast-like cell line COS-7^23^. The identification of MKK4 but not MKK7 as a downstream effector of LZK in our study may reflect an increased signaling specificity with endogenous substrates in a more relevant cell type. Further supporting cell type specificity in this signaling cascade, LZK activates endogenous MKK4 but not JNKs in HeLa cells of human cervical cancer origin (Fig. 2A).

Given the high sequence similarities between LZK and DLK, we next sought to compare their biochemical properties. We found that both proteins activate endogenous MKK4 but not MKK7 (among the MAP2Ks), and JNKs but not p38s or ERKs (among the MAPKs) (Fig. 2D,E), indicating similarity in their signaling capabilities. Interestingly, it was previously reported that DLK phosphorylates recombinant MKK7 but not MKK4 *in vitro*^28^, while MKK4 appears to be the main MAP2K acting downstream of DLK with MKK7 playing a minor role in the regulation of post-injury axon degeneration^29^. Together, these observations underscore context-dependent specificity in signaling, and additionally, suggest the requirement for cellular adaptor proteins in DLK/LZK signaling.

### Neuronal maturation and axonal outgrowth are accompanied by upregulation of LZK-MKK4-JNK signaling in cerebellar granule neurons in culture

**Figure 3:**
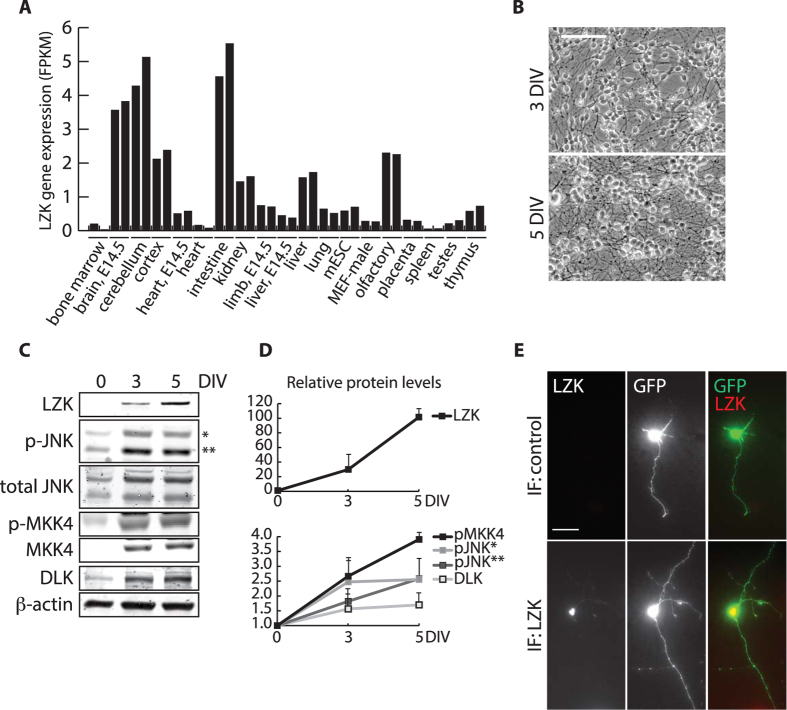
Neuronal maturation-dependent upregulation of LZK-MKK4-JNK in cerebellar granule neurons. (**A**) Graph compares gene expression of LZK in nineteen tissues from adult and embryonic (E14.5) mice based on published RNA-Seq dataset^30^. Each tissue sample was run in duplicates. LZK expression level is presented as fragments per kilobase of exon per million fragments mapped (FPKM). (**B**) Bright field images show axon growth of primary cerebellar granule neurons (CGNs) isolated from postnatal (P7) mice cultured for 3 and 5 days *in vitro* (DIV). Scale bar = 50 μm. (**C**) Total cell lysates from CGNs cultured for 0 (freshly dissociated cells before plating), 3, and 5 DIV were immunoblotted for the indicated endogenous proteins. *JNK 54 kDa isoform; **JNK 46 kDa isoform. (**D**) Based on (**C**) graphs show immunoblot signal-based quantification of endogenous LZK, p-JNK1/2, and p-MKK4 protein levels that were first normalized to β-actin in the corresponding samples, followed by subsequent normalization of this ratio on 3 and 5 DIV to that of 0 DIV (presented as baseline of 1 on graphs). *JNK 54 kDa isoform; **JNK 46 kDa isoform. (**E**) CGNs transfected with pBI empty vector expressing GFP for visualization of cell morphology were cultured for 3 DIV and immunostained for endogenous LZK (top panel), or with secondary antibody only as negative control (bottom panel). Scale bar = 50 μm.

To guide our functional analyses of LZK, we searched available gene expression datasets based on RNA Sequencing (RNA-Seq) of annotated genes in the mouse genome and identified a transcriptome analysis that is informative to determining LZK expression in various mouse tissues^30^. According to this RNA-Seq dataset, the mouse cerebellum is one of two tissues among all examined to have the highest LZK expression (Fig. 3A). *In situ* hybridization data on adult mouse brain from the Allen Brain Institute also indicate high level of LZK mRNA expression in the granule cell layer of the cerebellum (not shown). We thus focused our analyses of LZK in axon growth from primary neurons on cultured mouse cerebellar granule neurons (CGNs).

CGNs exhibit a high degree of polarization when cultured *in vitro* that allows morphology-based distinction between axons and dendrites^31^,^32^. As expected, mouse CGNs cultured from postnatal day 7 (P7) cerebellum exhibited steady axon outgrowth from seeding to 5 days *in vitro* (DIV) that accompanied neuronal maturation following isolation (Fig. 3B). During this time course, expression of endogenous LZK protein was initially below detection levels by immunoblotting, but increased to detectable levels by 3 DIV and continued to rise by 5 DIV, concomitant with an increase in the activation of endogenous MKK4 and JNKs (Fig. 3C,D). DLK, which is present in granule neurons in the developing and adult mouse cerebella^13^,^33^, also followed a similar trend of increase in expression over this time course (Fig. 3C,D). Immunofluorescence staining for endogenous LZK confirmed its expression mainly in the cell body of CGNs cultured for at least 3 DIV (Fig. 3E). This upregulation of the LZK-MKK4-JNK axis during the process of CGN neurite outgrowth is consistent with a possible role for LZK as a positive regulator of axon outgrowth.

### LZK overexpression enhances axon growth in mouse central nervous system neurons

**Figure 4:**
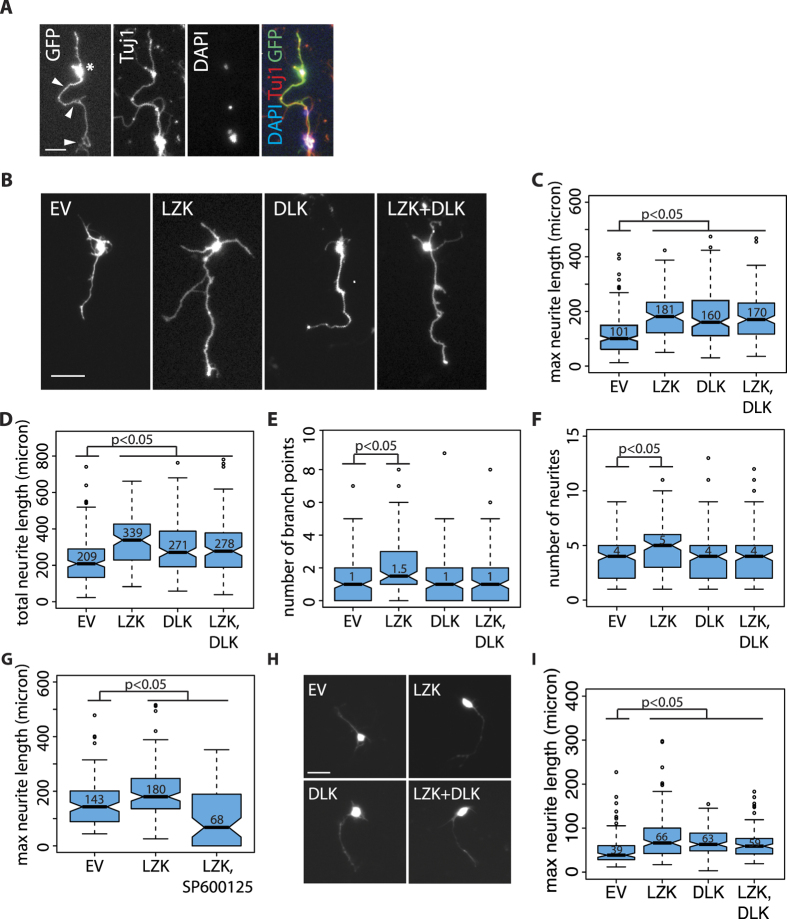
LZK overexpression enhances axon growth in mouse central nervous system neurons. (**A**) For *in vitro* axon growth assays of CGNs, axon lengths of GFP and TuJ1-double positive cells indicative of pBI expression and neuronal identity respectively were quantified. Measurement was based on GFP. Asterisk marks cell body; white arrowheads point to the longest axon. Scale bar = 20 μm. (**B**) CGNs from wild-type mice were transfected with the indicated pBI plasmids. Images show GFP-positive CGNs with maximum axon lengths representative of the median values in the corresponding conditions. Scale bar = 50 μm. (**C–F**) Boxplots quantify maximum axon lengths (**C**) total neurite lengths (**D**) number of branch points (**E**) and number of neurites per neuron (**F**) in CGNs transfected with the indicated pBI vectors. For all boxplots, median values for each condition are shown within graphs. EV indicates empty vector. All boxplot edges extend to the 25^th^ and 75^th^ percentiles; whiskers extend to non-outliner extremes; points beyond whiskers represent outliners. *p*-values by Wilcoxon test; n = 120–160 neurons per condition. (**G**) Boxplot quantifies maximal axon growth of P7 CGNs treated with 25 μM JNK inhibitor SP600125 upon transfection with the indicated pBI vectors. EV indicates empty vector. (**H**) Hippocampal neurons isolated from wild-type postnatal day 6 (P6) mice were transfected with the indicated pBI plasmids. EV is empty vector negative control. Images show GFP-positive hippocampal neurons indicative of transfection with pBI vectors. Scale bar = 20 μm. (**I**) Boxplot quantifies maximum axon lengths in hippocampal neurons transfected with the indicated pBI vectors shown in (**H**).

The below-detection levels of endogenous LZK protein expression in CGNs before 3 DIV offered a time window to test the effect of LZK overexpression on axon growth with minimal interference from endogenous LZK. CGNs were transiently transfected with pBI-LZK coexpressing GFP 18 hours after plating, followed by fixation 24 hours later. For comprehensive assessment of the effects of LZK overexpression on axon growth, parameters including axon length, branching, and total number of neurites of GFP and TuJ1 double-positive cells indicative of expression of transfected pBI vectors and neuronal identity, respectively, were measured based on GFP (Fig. 4A). GFP-positive CGNs from each experimental group with maximum axon lengths representative of the median values are shown in Fig. 3B. Compared to the control, exogenous LZK significantly increased the median maximum axon length by ~80% (Fig. 4C) and total neurite length by ~60% (Fig. 4D). Furthermore, LZK overexpression increased the number of branch points and neurites (Fig. 4E,F). Inhibition of JNKs, downstream effectors of LZK, by SP600125 abolished the axon growth-enhancing effects of LZK overexpression, indicating that JNK activity is required for the biological effect of LZK overexpression (Fig. 4G). The observation that SP600125 reduced axon growth below the level of control may reflect the role of JNKs in mediating signaling from endogenous LZK, endogenous DLK (Fig. 2D, 3C, 3D) and possibly other upstream regulators in addition to exogenous LZK. In comparison to LZK, DLK overexpression also increased axon length albeit to a slightly lesser extent (Fig. 4B–D), indicating that under comparable cultured conditions LZK and DLK display similar activities in promoting axon outgrowth. However, DLK overexpression had no effect on branching or number of neurites (Fig. 4E,F). Combined LZK and DLK overexpression did not additively or synergistically increase axon length compared to either overexpression alone (Fig. 4B–D), suggesting a ceiling effect of overexpression on axon growth and/or that co-overexpressed LZK and DLK signal through a common downstream pathway to regulate axon extension. Importantly, the growth promoting effect of LZK overexpression is not limited to CGNs. Overexpression of LZK, DLK, or both increased the median axon length in primary mouse hippocampal neurons by ~50–70% (Fig. 4H,I), as well as in cortical neurons by ~30–50% (Fig. S1A,B). Taken together, these observations indicate that both LZK and DLK enhance axon extension, but suggest that only LZK promotes branching and therefore the total number of neurites.

### Genetic depletion of LZK impairs JNK activation and axon growth in mouse central nervous system neurons

**Figure 5:**
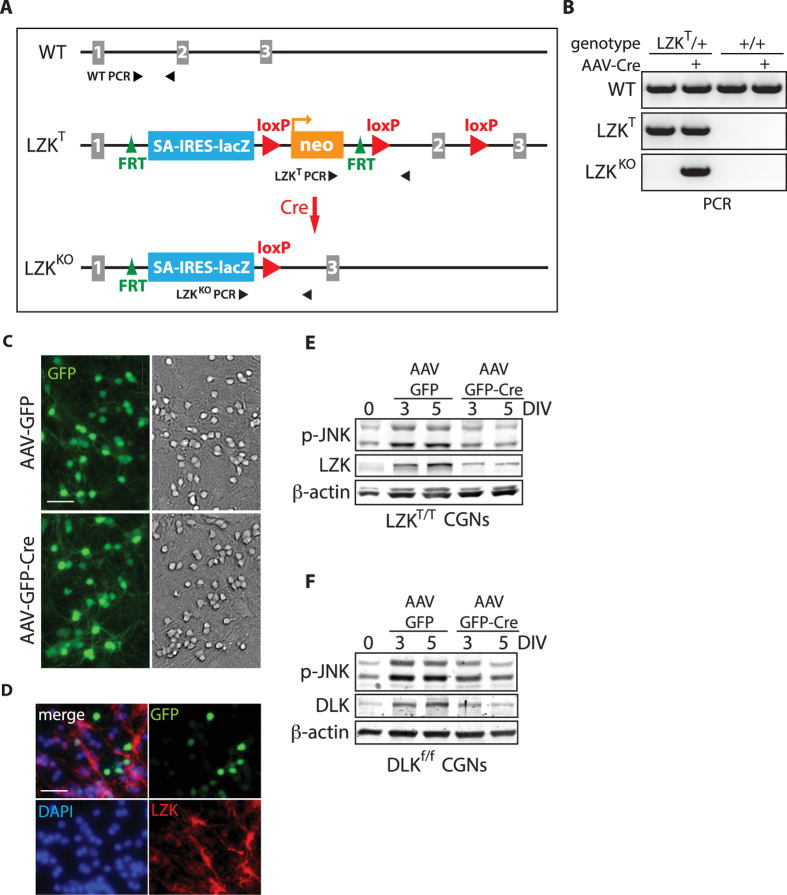
Generation and validation of LZK mutant mice and impaired JNK activation in LZK mutant CGNs. (**A**) Illustration of the LZK wild-type (WT), targeted (LZK^T^) and null/knockout (LZK^KO^) alleles. The first three exons of *LZK* are shown as grey rectangles. *FRT*, flippase (FLP) recognition site; SA, *En-2* gene splice acceptor; IRES, internal ribosomal entry site. The LZK^T^ allele contains three loxP sites, two of which flank exon 2, with the third upstream of β-actin promoter driven neomycin resistance gene (neo). Efficient Cre-mediated recombination between *loxP* sites would excise neo and exon 2 to create a null LZK^KO^ allele. Pairs of small black arrowheads indicate primer pairs for genotyping PCR. Allelic elements are not drawn to scale. (**B**) Validation of Cre-dependent conversion of LZK^T^ to LZK^KO^ allele *in vitro* by PCR. Primary CGNs isolated from mice of the indicated genotype were left untreated or treated with AAV-Cre. Genomic DNA was isolated from each condition and subjected to PCR targeting the WT, LZK^T^ or LZK^KO^ allele. (**C**) CGNs from LZK^T/T^ mice were infected with AAV-GFP or AAV-GFP-Cre 40 hours after plating and fixed on 5 DIV. (**A**) Based on the ratio of GFP-positive CGNs to total number of CGNs in bright field microscopy, infection efficiency of AAV-GFP and AAV-GFP-Cre are 64 and 63% respectively. Scale bar = 20 μm. (**D**) Visualization of LZK depletion in AAV-GFP-Cre infected CGNs by immunofluorescent staining of endogenous LZK. Scale bar = 20 μm. (**E**) CGNs purified from LZK^T/T^ mice were infected with control AAV-GFP or AAV-GFP-Cre to deplete LZK. Total cell lysates were collected on the indicated days *in vitro* (DIV) and immunoblotted for the indicated proteins. (**F**) CGNs from DLK^f/f^ mice were infected with control AAV-GFP or AAV-GFP-Cre to deplete DLK. Total cell lysates were collected on the indicated days *in vitro* (DIV) and immunoblotted for the indicated proteins.

To address the critical question of whether endogenous LZK is important for axon growth in primary CGNs, we turned to the use of targeted LZK mutant mice for loss-of-function analyses. Use of LZK mutant CGNs would allow direct assessment of the contribution of endogenous LZK to axon outgrowth as it circumvents possible off-target effects often associated with RNAi-mediated gene knockdown. We generated LZK mutant mice from embryonic stem cells carrying the Map3k13^tm1a(KOMP)Wtsi^ allele (referred to as LZK^T^, standing for LZK Targeted allele) obtained from the Knockout Mouse Project (KOMP) (see Materials and Methods for details). The LZK^T^ allele contains a LacZ reporter cassette, three *loxP* sites and a neomycin resistance gene (*neo*) upstream of exon 2 of the *MAP3K13 (LZK*) gene (Fig. 5A). Two of the three *loxP* sites flank exon 2 that encodes the first 55 amino acid residues of the kinase domain, including residue K195 essential for kinase activity, with the third *loxP* site upstream of *neo*. Exposure to Cre would excise *neo* together with exon 2, leading to a frame shift mutation that results in a null allele, herein referred to as LZK^KO^ (Fig. 5A).

To validate Cre-dependent conversion of LZK^T^ to the predicted null allele *in vitro*, CGNs from wild-type control or heterozygous LZK^T^ (LZK^T^/+) mice were infected with AAV-Cre (adeno-associated virus carrying a Cre expression construct), followed by genomic DNA isolation and subsequent PCR amplification targeting the wild-type, LZK^T^, or LZK^KO^ allele (see PCR primer positions in Fig. 5A). Indeed, infection with AAV-Cre in LZK^T^/+ CGNs led to the genomic excision event predicted for the LZK^KO^ allele (Fig. 5B). As a control, only the targeted LZK^T^ allele but not the LZK^KO^ allele was detected by genomic PCR in uninfected LZK^T^/+ CGNs (Fig. 5B). To assess the efficiency of Cre-mediated LZK knockout, we infected primary CGNs isolated from homozygous LZK^T/T^ mice on 2 DIV with AAV-GFP-Cre and observed a ~60% infection efficiency as early as three days post-infection in CGNs based on the percentage of GFP-positive cells in the total cell population (Fig. 5C). Immunofluorescence staining for endogenous LZK showed absence of LZK expression in GFP-positive CGNs infected with AAV-GFP-Cre (Fig. 5D). Western blot analyses indicated that AAV-GFP-Cre consistently reduced endogenous LZK protein levels in LZK^T/T^ CGNs to ~40% of the control levels (Figs 5E and 7B,D). Together, these data suggest a near complete knockout of LZK in ~60% of the cells infected.

To evaluate the contribution of LZK to JNK activation over the course of neuronal maturation *in vitro* (Fig. 3B,C), LZK^T/T^ CGNs were infected by AAV-GFP-Cre and grown for five days. LZK depletion partially reduced JNK activation by ~30% on average on 5 DIV (Fig. 5E). For comparison, we also evaluated DLK using a similar genetic depletion approach. As with LZK, Cre-mediated DLK knockout in CGNs from a DLK conditional mutant (DLK^f/f^) mouse line also impaired JNK activation (Fig. 5F).

**Figure 6:**
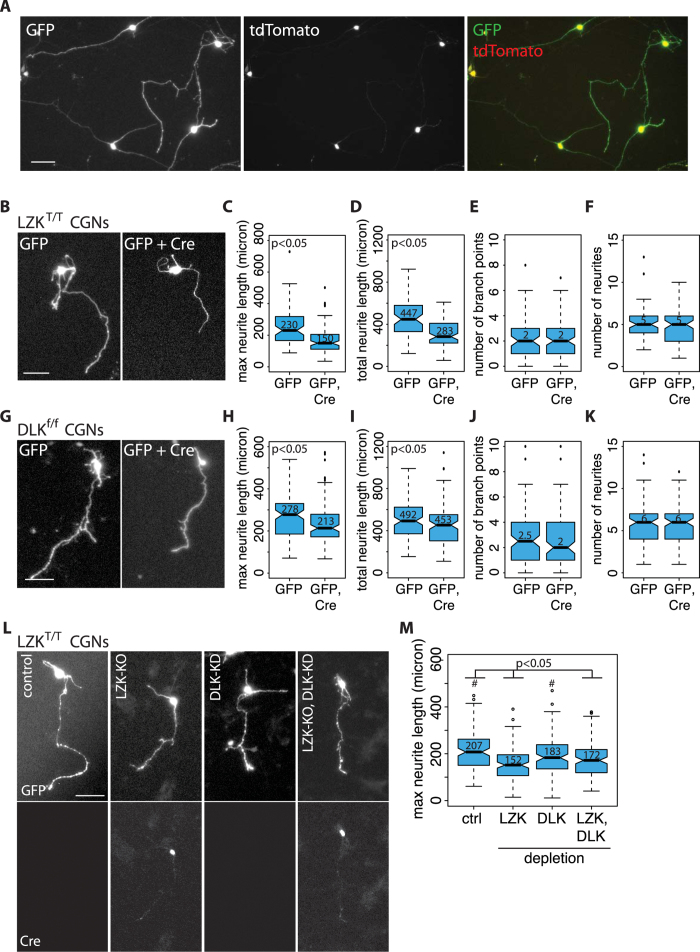
LZK depletion impairs axon growth of cerebellar granule neurons isolated from LZK^T^ mice. (**B**) CGNs from LZK^T/T^ mice were co-transfected with pMAX-GFP and pCAG-Cre at 1:3 ratio to generate GFP-labeled LZK^KO/KO^ CGNs. Images show GFP-positive CGNs with maximum axon lengths representative of the median values in the corresponding conditions. Scale bar = 50 μm. Boxplot quantifies maximum axon lengths (**C**) total neurite lengths (**D**) branching (**E**) and total number of neurites (**F**) in LZK^T/T^ CGNs co-transfected with the indicated vectors. Median values are shown for each condition within the graph. (**G**) CGNs from DLK^f/f^ mice were co-transfected with pMAX-GFP and pCAG-Cre as in (**B**). Images show GFP-positive CGNs with maximum axon lengths representative of the median values in the corresponding conditions. Scale bar = 50 μm. Boxplot quantifies maximum axon lengths (**H**) total neurite lengths (**I**) branching (**J**) and total number of neurites (*K*) of DLK^f/f^ CGNs co-transfected with the indicated vectors. Median values are shown for each condition within the graphs. n > 100 neurons per condition. (**L**) LZK^T/T^ CGNs were transfected with pGIPZ empty plasmid (control), pGIPZ and Cre (LZK-KO), pGIPZ-DLK-shRNA (DLK-shRNA), or Cre and pGIPZ-DLK-shRNA (LZK-KO, DLK-shRNA). (*Left*) pGIPZ plasmids co-express GFP to fluorescently label transfected cells. Cre expression was visualized by immunofluorescence staining. GFP-positive CGNs with maximum axon lengths representative of the median values in the corresponding conditions are shown. Scale bar = 50 μm. Quantification of maximum axon lengths shown in (**M**). Median values are shown for each condition within the graphs. All boxplot edges extend to the 25^th^ and 75^th^ percentiles; whiskers extend to non-outliner extremes; points beyond whiskers represent outliners. *p*-values by Wilcoxon test, n > 100 neurons per condition.

To conduct LZK loss-of-function analysis on axon outgrowth, we isolated CGNs from postnatal homozygous LZK^T/T^ mice and transiently co-transfected these cells with a Cre-expressing plasmid or an empty control vector along with a GFP-expressing plasmid (3:1 ratio) to label transfected cells. This method achieved nearly 100% co-transfection efficiency, as assessed by co-transfection of GFP- and tdTomato-expressing plasmid (Fig. 6A). We used this co-transfection method because it allows for a more rapid Cre expression and consequently LZK knockout than AAV-based gene delivery. Following co-transfection, GFP and Cre co-transfected LZK^T/T^ cells displayed a ~35% reduction in maximum axon length and ~40% reduction in total neurite length (Fig. 6B–D). This analysis provides strong support that endogenous LZK positively regulates axon extension. No effect on branching or total number of neurites was observed (Fig. 6E,F), suggesting that other genes might compensate for the loss of LZK in regulating axonal branching. In comparison, genetic depletion of DLK in DLK^f/f^ CGNs reduced axon growth by ~10–25% (Fig. 6G–I), with no significant changes in other parameters of axon growth (Fig. 6J,K). Next we combined Cre-mediated LZK-knockout and shRNA-mediated DLK-knockdown in LZK^T/T^ CGNs to test the effect of depleting both LZK and DLK on axon outgrowth. LZK or DLK depletion alone reduced axon length by ~30% and ~10% respectively (Fig. 6C,H), whereas double depletion resulted in an intermediate ~20% reduction (Fig. 6L,M). Thus, of the two kinases, both gain- and loss-of-function approaches suggest a greater effect of LZK manipulation on axon growth and there does not appear to be any additive or synergistic effect of manipulating both in the CGNs.

To extend LZK loss-of-function analyses to another CNS neuronal type, we examined cortical neurons using an *in vitro* microfluidic chamber-based axotomy and axon regeneration assay^34^. In this approach, E18.5 cortical neurons were seeded in the soma compartment and allowed to grow axons through microgrooves into the axonal compartment over seven days. On 7 DIV, neurons were transfected with control siRNA or a pool of four LZK-siRNAs in the soma compartment. Axons were cut by vacuum aspiration in the axonal compartment on 8 DIV, and axon regeneration was assessed 24 h after axotomy. siRNA-mediated knockdown of LZK significantly reduced axon regeneration from cortical neurons using two different measures (Fig. S1C,E). These data extend the results from CGNs above and further indicate that LZK promotes axon growth of another neuron type after injury with an *in vitro* axotomy model.

### LZK-dependent JNK activation in response to neuronal activity deprivation in cerebellar granule neurons

Primary murine CGNs cultured *in vitro* undergo rapid morphological and biochemical differentiation to acquire neuronal properties including neuronal polarization, voltage-dependent sodium and calcium channels, neurotransmitter receptors, and stimulus-coupled glutamate release^35^,^36^,^37^. This culture paradigm has been used to model *in vivo* molecular events of granule cell biology. For example, modulation of extracellular potassium chloride (KCl) concentration is routinely used to examine activity-dependent responses in cultured CGNs^31^,^36^,^38^,^39^. Maintaining dissociated CGNs at elevated KCl (at least 20 mM) simulates an electrically active state, as depolarization leads to calcium influx that recapitulates the effects of physiological stimulation via excitatory neurotransmitter receptors^36^. In contrast, KCl deprivation in cultured CGNs creates a non-depolarized state that silences neuronal activity and simulates deafferentation^39^,^40^,^41^. Whereas rat CGNs undergo apoptosis upon KCl withdrawal, mouse CGNs survive^42^.

**Figure 7:**
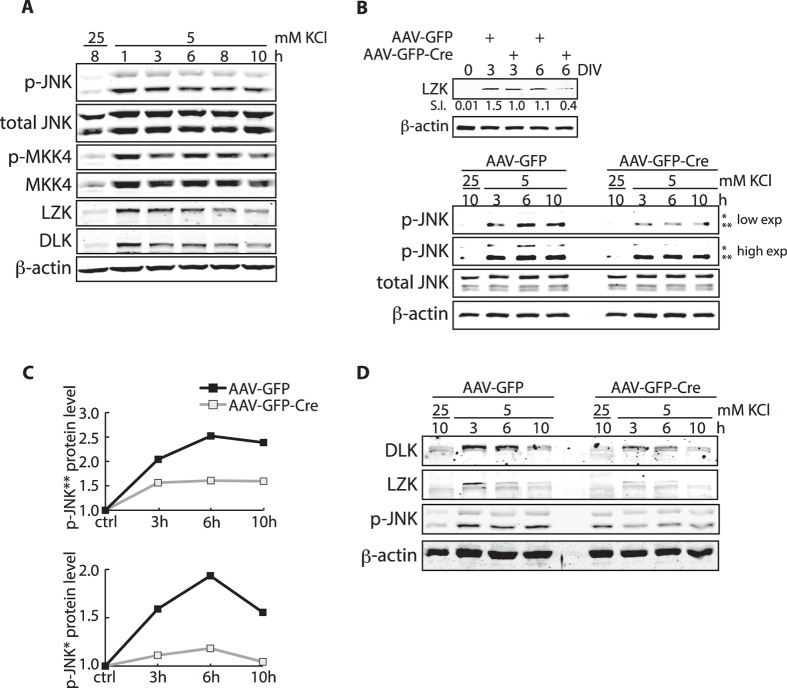
Silencing of neuronal activity by potassium withdrawal leads to LZK-dependent activation of JNKs in cerebellar granule neurons. (**A**) CGNs from wild-type mice were maintained in media containing 25 mM KCl for five days. Potassium withdrawal was performed by switching to media containing 5 mM KCl without serum as previously described^39^ on day 6. CGNs were collected at the indicated times following potassium withdrawal and total cell lysates were immunoblotted for the indicated endogenous proteins. (**B**) LZK^T/T^ CGNs were infected with AAV-GFP or AAV-GFP-Cre 40 hours after plating. *Top panel,* total cell lysates collected on 0, 3, 6 DIV were immunoblotted for endogenous LZK to assay LZK depletion in the total cell population. Immunoblot-based quantification of signal intensity (S.I.) of endogenous LZK protein levels normalized to that of β-actin is shown. *Bottom panel*, AAV-GFP or AAV-GFP-Cre infected LZK^T/T^ CGNs were subjected to potassium withdrawal on 6 DIV. Total cell lysates collected at the indicated times following treatment were immunoblotted for the indicated endogenous proteins. Two different exposures (low and high) for p-JNK2 and p-JNK1 are shown for better comparison. *JNK 54 kDa isoform; **JNK 46 kDa isoform. (**C**) Graphs quantify endogenous p-JNK protein levels from the bottom panel in (**B**). Immunoblot-based quantification of JNK protein expression was first normalized to β-actin in the corresponding sample, and shown as a value relative to the control (presented as baseline of 1). (**D**) LZK^T/T^ CGNs were infected with control AAV-GFP or AAV-GFP-Cre 40 h after plating. AAV-infected LZK^T/T^ CGNs were subjected to potassium withdrawal on 6 DIV. Total cell lysates collected at the indicated times following treatment were immunoblotted for the indicated endogenous proteins.

Because JNKs, identified here as *in vivo* downstream effectors of LZK in neurons, have been described as key mediators of neuronal response to KCl withdrawal in rodent CGNs^39^, we sought to determine (1) whether KCl withdrawal – a condition that deprives neuronal activity as occurs following axotomy of the peripheral branch of dorsal root ganglion neurons^43^ – activates the endogenous LZK signaling cascade; and (2) to what extent JNK activation is dependent on LZK in this response with our culture system. We cultured mouse CGNs in complete media containing 25 mM KCl, then switched to media with 5 mM KCl on day 6 *in vitro*. Over the time course of 10 hours following KCl withdrawal, we observed a rapid increase in endogenous LZK at the protein level accompanied by activating phosphorylation of both MKK4 and JNKs as early as 1 hour after treatment, and this increase was maintained for at least 10 hours (Fig. 7A). Consistent with our finding that LZK and DLK share the downstream MKK4-JNK axis, endogenous DLK protein levels also increased (Fig. 7A). Upregulation of total MKK4 and JNK protein expression likely contributed to their enhanced activation (Fig. 7A).

Given the upregulation of the LZK-MKK4-JNK axis after KCl deprivation, we next assessed the contribution of LZK to this response. CGNs from LZK^T/T^ mice were infected *in vitro* with control AAV-GFP or AAV-GFP-Cre to generate LZK-null CGNs (Fig. 7B). As described above, at ~60% infection efficiency, depletion of LZK protein was achieved consistently at ~60% in LZK^T/T^ CGNs (Fig. 7B, *Top* panel). In LZK-depleted cells, activation of JNKs was diminished by ~40% at the height of the response to KCl withdrawal occurring at 6 hours post-treatment (Fig. 7B–D). This partial impairment of JNK activation may be the result of LZK knockout in only ~60% of the whole cell population and/or partial functional redundancy with DLK and possibly other MAP3K in this response. Interestingly, in addition to reducing JNK activation upon potassium deprivation, LZK depletion also diminished DLK protein levels (Fig. 7D), possibly due to decreased JNK activity under these conditions, as JNK-dependent phosphorylation of DLK promotes DLK stability^44^. Taken together, these findings show that KCl withdrawal, a culture condition causing neuronal activity deprivation in CGNs, leads to LZK-dependent activation of its downstream signaling.

Upregulation of LZK-MKK4-JNK at the level of protein expression and activation following either dissociation of CGNs (Fig. 3C,D) or neuronal activity deprivation (Fig. 7A) suggests neuronal insult-dependent regulation and functions of LZK. Although LZK is expressed in the cerebellum in mice and humans^1^,^30^ (Fig. 3A), we did not observe any overt developmental defect in cerebellar formation in adult LZK knockout (LZK^KO/KO^) mice (data not shown). LZK knockout mice are also viable, fertile and grossly indistinguishable from their wild-type or heterozygous littermates. Lack of a prominent role in neuronal development may suggest a more dedicated physiological function for LZK following neuronal injury or insult, an intriguing possibility that remains to be fully explored.

## Discussion

LZK and DLK are both mammalian homologues of invertebrate DLK-1/Wallenda, which has been established as a key regulator of various aspects of nervous system development and physiology ranging from synapse formation to neuronal and axonal responses to insult or injury^4^,^5^,^7^,^8^,^9^,^16^. In spite of major advances in understanding the role of DLK in the mammalian system, the functions and regulation of LZK remain surprisingly poorly understood. Our study provides the first evidence that LZK promotes axon outgrowth of mammalian central nervous system (CNS) neurons in a cell-autonomous manner. Additionally, we identified MKK4 and JNKs as endogenous effectors of the LZK signaling pathway in neurons that is activated under conditions simulating neuronal activity deprivation that may favor regeneration. Thus, LZK may act as a sensor of neuronal insult that, depending on the cellular context, can promote axon growth. Our data provide the rationale to pursue the *in vivo* roles of LZK after axonal injury in future studies. In this context, it would be of interest to dissect the interaction among LZK/DLK, MKK4/7 and JNKs in neuronal responses to axonal injury. A better understanding of neuronal responses to injury including axonal regrowth is important to the development of therapeutic strategies to promote recovery from CNS injury^45^.

In characterizing the biological and signaling activities of LZK, we observed that LZK signals through MKK4 and JNKs in N2a and primary CGNs, both neuronal cell types in which LZK promotes neurite/axon growth. In contrast, LZK activates only MKK4, but not JNKs, in HeLa cells of human cervical cancer origin. This suggests that JNKs mediate LZK signaling in a cell type-dependent manner, which, in neurons, leads to axon growth. Indeed, JNKs are required for axonogenesis, where activated JNKs are enriched in axons, and for axon regeneration^46^,^47^,^48^. JNKs have been described to exert their function on axon extension by activating c-Jun and activating transcription factor 3 (ATF3) in the nucleus^47^; alternatively, JNKs may regulate microtubule dynamics in the cytoplasm^48^,^49^,^50^,^51^. The extent to which these mechanisms contribute to LZK-mediated axon growth remains to be explored. In *C. elegans*, DLK-1 signals predominantly through p38 to control axon growth^4^,^7^,^8^, suggesting evolutionary drift in MAPK function in the LZK/DLK signaling pathway.

In addition to delineating the LZK signaling cascade, we identified two activating conditions of the endogenous LZK-MKK4-JNK pathway in primary CGNs. The first is during the natural process of axon growth during *in vitro* neuronal maturation of CGNs isolated from early postnatal mice. This is consistent with our finding that overexpression of LZK early in culture promotes axon growth in these cells. The second is potassium withdrawal, which deprives neuronal activity. Interestingly, it has previously been shown that electrical silencing following a peripheral lesion of sensory axons is important for axon regeneration^43^. LZK-dependent activation of JNKs under conditions mimicking electrical silencing, together with our *in vitro* evidence supporting LZK as a positive regulator of axon growth, implicates a role for LZK in mediating neuronal response to upstream changes in the electrical state with downstream effects on axon growth.

Our comparison of LZK and DLK revealed that their signaling pathways converge at MKK4-JNK in the MAPK cascade, that both kinases and downstream MKK4-JNK are upregulated gradually during the extended time of axon growth as well as acutely following neuronal activity deprivation in cultured cerebellar granule neurons. In terms of their neuronal functions in axon growth, LZK and DLK similarly promote axon extension in CGNs and hippocampal neurons. Notably, co-overexpression of both kinases did not synergistically enhance axon length compared to overexpression of either kinase alone. Given that LZK and DLK activate common downstream effectors in neurons based on our observations and can exist as homo- or hetero-dimers/oligomers based on reported findings^3^,^23^,^52^, the lack of synergistic effects on axon growth suggests that LZK and DLK homo- or hetero-dimers/oligomers activate a common downstream pathway to promote axon growth. Consistent with the role of these two kinases as positive regulators of axon growth, depletion of either kinase reduced axon length. However, combined depletion of both kinases did not further decrease axon length compared to individual depletion. This may be the result of positive cross-regulation between LZK and DLK, such that downregulation of one kinase represses the activity of the other. Supporting this idea is evidence that JNKs, their common downstream effectors, feedback positively on the protein levels of DLK^44^ and LZK (Fig. 2C). Such cross-regulation between LZK and DLK adds complexity to the dissection of their respective contributions, *in vitro* and *in vivo*. Additionally, in comparing the effects of LZK and DLK on axon branching, only LZK overexpression increased branching and correspondingly the total number of neurites. This suggests that LZK regulates aspects of axon growth and morphology such as the initial formation of new axonal sprouts in addition to neurite length, a function not shared with DLK.

While LZK and DLK exhibit mostly parallel properties and similar modes of regulation under the conditions examined here, it remains possible that differential temporal and spatial regulation of their expression in other biological processes confers these kinases distinct signaling and functional specificities. In this regard, our observation that depletion of LZK alone can attenuate both axon growth of cerebellar granule neurons and KCl withdrawal-induced activation of downstream effectors strongly suggests that LZK and DLK serve overlapping but not completely redundant functions.

The unexpected cell type-specificity in signaling effectors downstream of LZK reported here parallels its cell type-specific function. Whereas we found that LZK promotes axon growth of N2a cells, CGNs, and hippocampal neurons, a previous study implicates LZK as a negative regulator of axon growth of cortical neurons by mediating the inhibitory effect of the myelin-associated inhibitor Nogo^24^. These apparently contradictory roles of LZK are reminiscent of the reported disparate activities of DLK in apoptosis, axon degeneration and axon regeneration^6^,^18^,^19^,^20^,^21^. In this regard, our finding is more in line with the recent report that LZK can functionally complement dlk-1 mutants in *C. elegans*^53^, supporting evolutionarily conserved roles for worm DLK-1 and mammalian LZK. The biological functions of LZK are just beginning to be revealed and further understanding of its role in neuronal responses to injury will require a thorough understanding of the specific cellular and environmental contexts and the corresponding signaling pathways involved. In conclusion, our study established an axon growth-promoting role of LZK, defined its signaling axis in neurons, and revealed functional commonalities and cross-regulation between LZK and DLK. The possibility that two MAP3Ks coordinately regulate axon growth responses to neuronal injury in the mammalian CNS will have important implications on developing therapeutic strategies to promote axonal repair after CNS injury.

## Materials and Methods

### Cell culture, transfection and neurite growth assay

HeLa, N2a, and primary neuronal cultures were maintained at 37 °C with 5% CO_2_. HeLa and N2 were grown in DMEM with 10% fetal bovine serum (FBS). Transfection of HeLa and N2a cells were performed using jetPRIME reagent (Polyplus Transfection) following manufacturer’s protocol.

Primary cerebellar neurons collected from postnatal day 7 (P7) mice were cultured as described previously with modifications^31^,^39^. Briefly, P7 mouse cerebella were dissected, their meninges removed, and minced with razor blade. Dissociated tissue was then washed in HBSS (Life Technologies) followed by incubation with Accutase solution (EMD Millipore) at 37 °C for 15 min. Tissues were washed three times in HBSS to remove Accutase, followed by mechanical trituration in Neurobasal-A medium (Life Technologies) containing 10% FBS. Cell suspension was passed through a cell strainer with 40 μM nylon mesh (Fisher Scientific) to remove aggregates. Granule neurons were then plated on tissue culture plates coated with 0.001% poly-ornithine (Sigma) and 5 μg/ml laminin (Sigma) in Neurobasal-A medium containing 10% FBS, 2% B27 (Life Technologies), 0.5 mM L-glutamine, 100 U/ml penicillin and streptomycin (Life Technologies), and 25 mM KCl. Granule neurons were seeded in 12-well tissue culture plates at 1–1.5 × 10^6^ cells per well for biochemical assays, or 0.2–0.25 × 10^6^ cells per well for neurite growth. AraC was added at final concentration of 3 μM ~48 hours after seeding to inhibit growth of non-neuronal cells; glucose was added at a final concentration of 25 mM 72 hours after seeding. Primary cerebellar granule neurons were transfected by calcium phosphate as previously described^54^ with 2X HEPES-buffered saline (280 mM NaCl, 10 mM KCl, 1.5 mM Na_2_HPO_4_, 12 mM dextrose, 50 mM HEPES, pH 7.05) and 2 M CaCl_2_. AAV infection was performed at 10,000 multiplicity of infection. KCl withdrawal was performed as previously described on day 6 in culture^39^.

Primary hippocampal neurons collected from postnatal day 6 (P6) mice were cultured as described previously with modifications^55^. Briefly, P7 mouse hippocampi were dissected in Hibernate^®^-E supplemented with 2% B-27^®^ Serum-Free Supplement and 0.5 mM GlutaMAX™, their meninges removed, and minced with razor blade. Dissociated tissue was then washed in Hibernate-E medium without Ca^2+^ followed by a 30 min incubation in Papain solution (2 mg/ml, Worthington) at 37 °C. Tissues were washed with Hibernate-E -2% B-27-GlutaMAX twice, then gently triturated with fired-polished Pasteur pipette. Suspension was filtered through a cell strainer with 40 μM nylon mesh (Fisher Scientific) to remove aggregates and applied to an OptiPrep density gradient. After centrifugation, the neuronal enriched fraction was collected, washed with Hibernate-E −2% B-27-GlutaMAX, and plated on tissue culture plates coated with poly-ornithine in Neurobasal-A medium containing 10% FBS, 2% B27 (Life Technologies), 0.5 mM L-glutamine, 100 U/ml penicillin and streptomycin (Life Technologies), and 25 mM KCl. Hippocampal neurons were seeded at 2.5 × 10^4^ cells per well in a 48-well plate for neurite growth. Cells were transfected the next using Lipofectamine-2000 (Life Technologies) as described by manufacturer recommendations, using 0,75 ug of DNA/well.

N2a cells were seeded in 24-well tissue culture plates at 0.04 × 10^6^ cells per well. Primary cerebellar granule neurons were seeded in 12-well tissue culture plates at 0.2–0.25 × 10^6^ cells per well; hippocampal neurons were seeded in 48-well plates at 0.25 × 10^5^ cells per well. For gene overexpression assays: plasmid transfection was performed 16 hours after cell seeding and fixed in 4% paraformaldehyde 24 hours after transfection for immunofluorescence staining. For gene knockdown assays: N2a cells were fixed 48 hours after transfection; primary cerebellar neurons were infected with AAV 24–30 hours after seeding and fixed five days after infection for immunofluorescence staining. Images were captured using Zeiss Axio Observer.A1 microscope and maximum (longest) axon length per neuron was manually traced using NeuroJ plug-in on ImageJ-64. Maximum neurite lengths, total neurite lengths, number of branch points, and number of neurites per neuron were compared among experimental groups. Data was collected from 3–5 wells (100–200 neurons) per condition per experiment. Experiments were performed at least twice. Representative experiments are shown, with data showing similar trend collected from repeated experiments. Statistical analysis by Wilcoxon test.

Primary cortical neurons were collected from cortices of E18.5 mouse embryo brains. Briefly, after removal of meninges, cortices were collected in Hibernate-E supplemented with 2% B-27 Supplement and 0.5 mM GlutaMAX at 4 °C, followed by enzymatic digest in Hibernate-E medium without Ca^2+^ containing 2 mg/mL of sterilized papain for 30 minutes at 30 °C. Tissues were then washed in complete Hibernate-E medium and cells dissociated by trituration with fire-polished glass Pasteur pipettes in Neurobasal medium with 2% B-27, 2% FBS and 0.5 mM GlutaMAX. 10^5^ cells were plated in poly-D-lysine coated 24-well plates. Cells were transfected with Lipofectamine-2000 according to manufacturer’s instructions.

For microfluidic chamber experiments, microfluidic chambers (SND450 Xona Microfluidics) were placed on PDL coated 6-well plates and treated with UV light for 1 hour immediately before cells were plated. A total of 4 × 10^5^ cells in 15 μl media were seeded per chamber. Each chamber was filled with additional 90 μl media per site 2 h after seeding and the cells were kept in culture for 7 days to allow for axons to grow through microgrooves into the axonal compartment. On 7 DIV, cells were transfected with 5 nM siRNA (non-targeting control or pool of four LZK-specific siRNAs, see “siRNAs” section below for sequences) using Lipofectamine RNAiMAX (Thermo Fisher) according to manufacturer’s instructions. Axons were cut by vacuum aspiration on 8 DIV. Neurons and axons were PFA-fixed and stained for Tuj1 on 9 DIV. Images taken from experimental replicates (total of six microfluidic chambers per condition) were used for analyses of axon regeneration by ImageJ64 software. For quantification of axon number, regenerating axons crossing lines spaced 250 μm and 500 μm away from the lesion site were manually counted; n = 750–1200 per condition. For quantification of axon density, the axonal compartment was divided into three zones at 250 μm increments away from the lesion site, each zone with width of 1.5 mm. Integrated density of axons within each zone was automatically quantified.

### Expression plasmids

pCS2+-FLAG-LZK and pCS2+-FLAG-LZK-K195A were generous gifts from Dr. Anne Vojtek (University of Michigan, Ann Arbor, USA)^24^. Second ATG start codon downstream of FLAG was deleted using site directed mutagenesis from both plasmids. Mouse LZK and LZK-K195A (a 2880 bp insert with restriction sites for BamH1 on the 5′ end and Not1 on the 3′ end) were separately subcloned from pCS2+ plasmids into BamH1-NotI sites of pBI-CMV3 vector carrying ZsGreen, a green fluorescent protein derived from *Zoanthus sp.* reef coral (Clontech, cat # 631632). Mouse DLK (a 3602 bp insert with restriction sites for BamH1 on the 5′ end and Not1 on the 3′ end) was subcloned from pYX-Asc-DLK obtained from Invitrogen (Clone ID 6401321) into BamH1-NotI sites of pBI-CMV3 vector. pMAX-GFP was obtained from Polyplus Transfection. GIPZ-nonsilencing-shRNAmir control, pGIPZ-LZK-shRNAmir, and GIPZ-DLK-shRNAmir were purchased from GE Healthcare-Dharmacon (RHS4346, RMM4431-99010732, RMM4431-200416419, respectively). Mature antisense sequence of GIPZ-LZK-shRNAmir is ATCAGTGGAATATGCCTTC; antisense sequence of GIPZ-DLK-shRNAmir is TGATGATGACATCTTTCGG.

### siRNAs

ON-TARGET plus siRNAs were purchased from GE Healthcare-Dharmacon. Target sequence of non-targeting control siRNA (D-001810-01-05) is UGGUUUACAUGUCGACUAA; LZK-siRNA #1 (J-056427-05): CUAAGGAACUCAGUGAUAA; LZK-siRNA #2 (J-056427-06): GGAAGUGGACAGUGAAGUA; LZK-siRNA #3 (J-056427-07): CAGCAACCAUGCACAAAGA; LZK #4 (J-056427-08): GAACACGAACGGACCAGAA. Control or a pool of LZK-specific siRNAs was used at final concentration of 5 nM.

### AAV viruses

AAV2/8-GFP (titre 6.3 × 10^14^ GC/ml) and AAV2/8-GFP-IRES-Cre (titre 7.45 × 10^15^ GC/ml) were purchased from Boston Children’s Hospital Viral Core (Boston, USA). AAV2/2-Cre (titre 5.09 × 10^11^ GC/ml) was purchased from Gene Transfer, Targeting and Therapeutics Core at Salk Institute for Biological Studies (La Jolla, USA).

### Antibodies

DLK rabbit antisera were generated against a fusion protein between glutathione S-transferase (GST) and the C-terminal 223 amino acid residues of DLK/MAP3K12 as described^56^; LZK (chicken) antibodies were generated against a fusion protein between GST and the C terminal 185 amino acid residues of LZK/MAP3K13 (both made by L.B.H.’s lab, with the specificity of DLK antibodies verified using DLK mutant tissues provided by Itoh and DiAntonio labs). Commercially available antibodies used were: LZK (R06696; Sigma-Aldrich), FLAG (M2; Sigma-Aldrich), phospho-(Thr183/Tyr185)-JNK1/2 (G9, Cell Signaling Technology), JNK1/2 (56G8, Cell Signaling Technology), phospho-(Ser257)-MKK4 (C36C11, Cell Signaling Technology), MKK4 (rabbit, Cell Signaling Technology), phospho-(Ser271/Thr275)-MKK7 (rabbit, Cell Signaling Technology), MKK7 (rabbit, Cell Signaling Technology), phospho-(Thr180/Tyr182)-p38 (rabbit, Cell Signaling Technology), p38 (rabbit, Cell Signaling Technology), phospho-(Thr202/Tyr204)-ERK1/2 (D13.14.4E, Cell Signaling Technology), phosphor-(Ser63)-cJun antibody (rabbit, Cell Signaling Technology), TuJ1 (mouse, BioLegend), Cre (rabbit, Covance), β-actin (C4, Millipore).

### Immunofluorescence staining

Cells were grown on tissue culture plates and fixed in 4% paraformaldehyde in PBS at 25 °C for 15 min. After fixation, samples were permeabilized in 0.4% Triton X-100 and 3% BSA in PBS for 30 min, followed by blocking in 0.01% Triton X-100 and 5% BSA in PBS for 30 min. Samples were then incubated in primary antibody diluted in 0.1% Triton X-100 and 0.1% BSA in PBS overnight at 4 °C in humid chamber. Samples were subsequently subjected to three 5 min washes in 0.2% Triton X-100 in PBS, followed by secondary antibody incubation for 1–2 hours and DAPI staining for 15 min at 25 °C in humid chamber in the dark. Samples were washed three more times and kept in Fluoromount-G (Southern Biotech). Immunofluorescent analysis was conducted on Zeiss Axio Observer.A1 microscope. Images were captured and analyzed using AxioVision 4.7 and ImageJ-64 software.

### Immunoblotting

For Western blots of total cell lysates, cells were washed with cold PBS, detached from plates with a cell scraper, and lysed on ice for 20 min after resuspension in lysis buffer (50 mM Tris-HCl [pH = 7.5], 150 mM NaCl, 0.5% NP-40, 5 mM EDTA, 5 mM EGTA, 20 mM NaF, 100 μM sodium-orthovanadate, 2 mM β-glycerophosphate, 1 mM DTT, 1 mM PMSF, 4 mg/ml aprotinin, 100 μM leupeptin, and 2 mg/ml pepstatin A). After 20 min centrifugation (13,200 rpm, 4 °C), supernatants were saved and protein concentration was performed using Quick Start Bradford Dye Reagent (Bio-Rad). Lysates were resuspended in loading buffer and analyzed by SDS-PAGE and Western blotting.

### DLK and LZK mutant mice

DLK^f/f^ mice were made in Dr. Lawrence B. Holzman’s laboratory, the detailed description of which will be published elsewhere. Exon 2, which contains the ATG start codon, is floxed in the DLK^f^ allele. For generation of LZK mutant mice, embryonic stem cell line JM8A1.N3 derived from the C57BL/6N strain carrying the LZK mutant allele Map3k13^tm1a(KOMP)Wtsi^, herein referred to as LZK^T^ (LZK Targeted allele) was obtained from the Knockout Mouse Project (KOMP) Repository. See Fig. 4A for allelic design. Injection of targeted mouse embryonic stem cells into blastocysts from C57BL/6N females was performed at the Transgenic Mouse and Gene Targeting Core at the University of California, San Diego. Chimeric mice were bred to wild-type albino C57BL/6N strain (C57BL/6/BrdCrHsd-TyrC from Harlan Sprague Dawley) for germline transmission of LZK^T^ allele. Germline transmission in offsprings from chimera mating was indicated by coat color and the LZK^T^ allele identified by PCR (Fig. 4A,B). LZK knockout mice were generated by breeding LZK^T^/+ mouse to germline Cre-expressing mouse, followed by Cre removal with subsequent mating of offspring to wild-type C57BL/6N mouse. Mouse maintenance and experiments were carried out in accordance with animal care guidelines at UCSD. The Institutional Animal Care and Use Committee approved all animal protocols. Genotyping PCR primer sequences are as follows:

Wild-type LZK

Forward primer: ACATCTGGATCTGAAGACAGCCAGG

Reverse primer: AGGTGCGTTTTCATTCTTCTGGACC

LZK^T^

Forward primer: GGGATCTCATGCTGGAGTTCTTCG

Reverse primer: AGGTGCGTTTTCATTCTTCTGGACC

LZK^KO^ (generated from LZK^T^ after Cre-mediated excision of exon 2)

Forward primer: GCTACCATTACCAGTTGGTCTGGTGTC

Reverse primer: GGACTGGTTGTGTCACTTGAGATGC.

## Additional Information

**How to cite this article**: Chen, M. *et al*. Leucine Zipper-bearing Kinase promotes axon growth in mammalian central nervous system neurons. *Sci. Rep.* **6**, 31482; doi: 10.1038/srep31482 (2016).

## Supplementary Material

Supplementary Information
