# Supplementary material for: Leucine Zipper-bearing Kinase promotes axon growth in mammalian central nervous system neurons
Source: Sci Rep. 2016 Aug 11;6:31482. doi: 10.1038/srep31482 (PMC4980599; doi:10.1038/srep31482)
Supplement: Supplementary Information [file srep31482-s1.pdf]

Supplementary Information (SI)

Leucine zipper-bearing kinase promotes axon growth in mammalian central nervous system neurons

Meifan Chen, Cédric G. Geoffroy, Hetty N. Wong, Oliver Tress, Mallorie T. Nguyen, Lawrence B. Holzman, Yishi Jin and Binhai Zheng

SI FIGURE LEGENDS

Figure S1. *LZK enhances axon extension and regeneration of cortical neurons.* (*A*) Cortical neurons isolated from E18.5 wild-type mice were transfected with the indicated pBI plasmids. EV is empty vector negative control. Images show GFP-positive hippocampal neurons indicative of transfection with pBI vectors. Scale bar = 25 μm. (*B*) Boxplot quantifies the median values of total neurite lengths, of cortical neurons transfected with the indicated pBI vectors. Median values for each condition are shown within the graph. All boxplot edges extend to the 25th and 75th percentiles; whiskers extend to non-outliner extremes; points beyond whiskers represent outliners. p-values by Wilcoxon test; n > 100 neurons per condition. (*C*) Representative images of regenerating axons from cortical neurons axotomized *in vitro* using microfluidic chambers (MFCs). Control indicates transfection with non-targeting siRNA (control); LZK-KD indicates transfection with a pool of four LZK-siRNAs. Scale bar is 100μm. Horizontal lines represent distances 250μm and 500μm away from the lesion site. (*D*) Average number of regenerating axons passing lines 250μm or 500μm from lesion site in MFCs. n >780 axons per condition. p-values by student's t-test. Standard error from replicates graphed. (E) Average density of regenerating axons in zone 1 (0-250μm), zone 2 (250-500μm), and zone 3 (500-750μm) away from the lesion site in MFCs. Statistics done as in (*D*).

Figure S2. *JNK activity promotes LZK protein expression*. N2a cells were transfected with empty vector (EV) or FLAG-LZK and treated with JNK inhibitor SP600125 or p38 inhibitor SB203580 at the indicated doses at the time of transfection for 12h. Total cell lysates were immunoblotted for the indicated proteins. (*A*) p-cJun indicates phospho-(Ser63)-c-Jun. (*B*) p-P38 indicates phospho-(Thr180/Tyr182)-p38. Immunoblot-based quantification of signal intensity (S.I.) of protein levels normalized to that of β-actin is shown for FLAG and p-cJun.

S1A

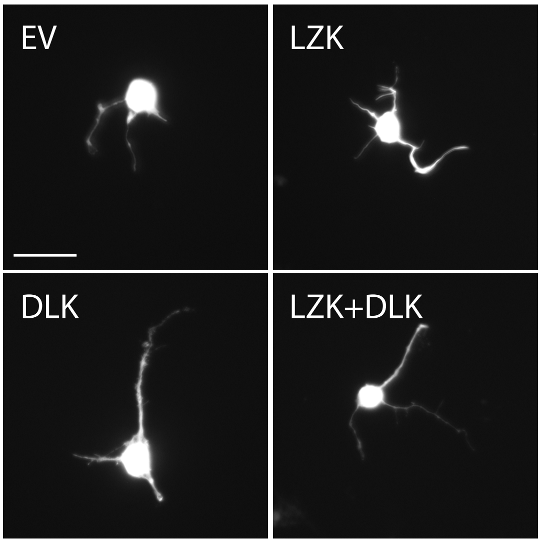

S1B

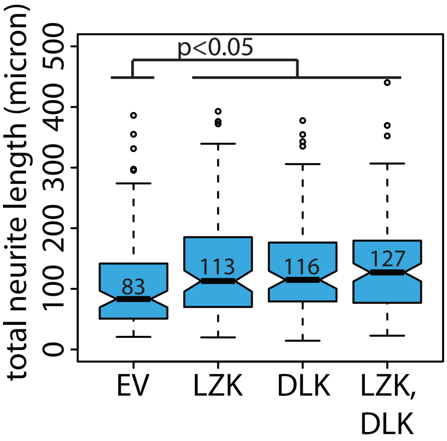

S1C

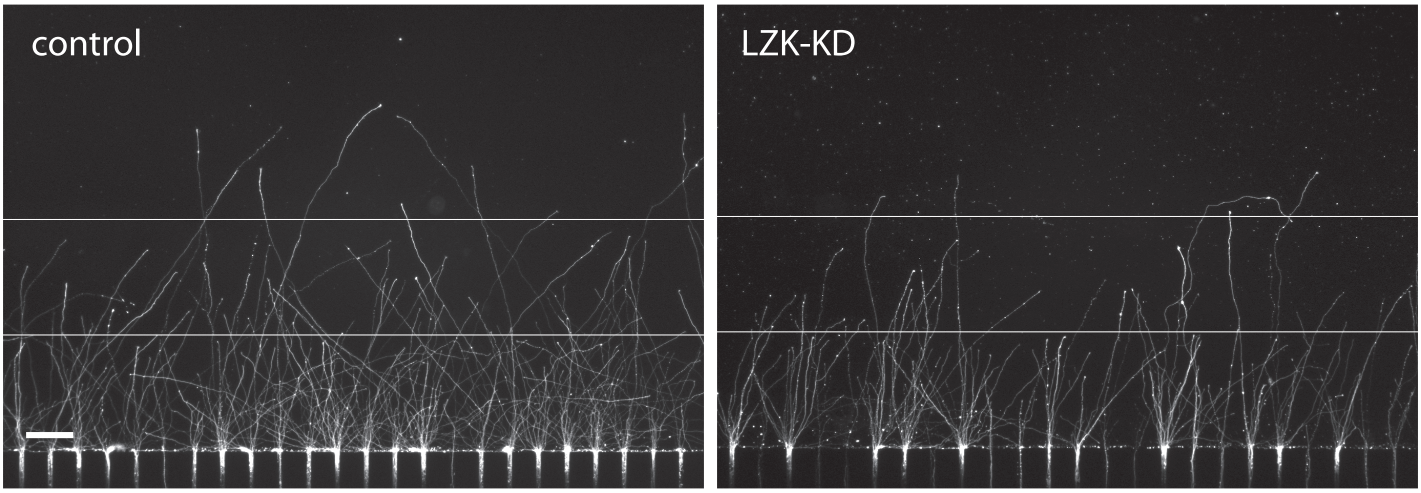

S1D

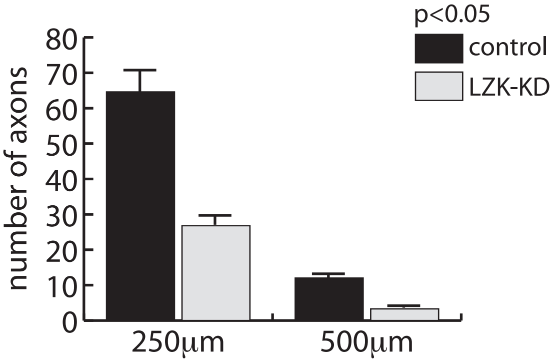

S1E

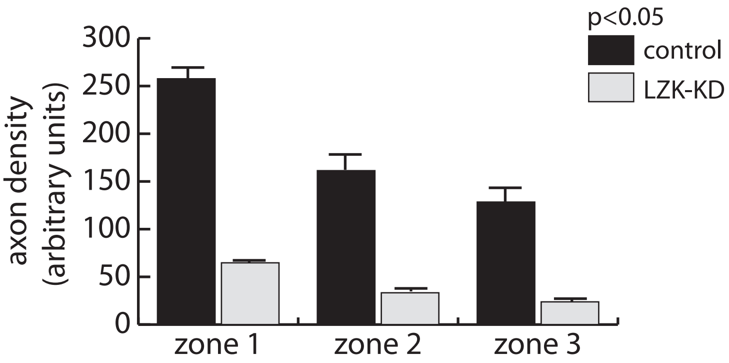

Chen *et al*, Supplemental Figure S1

S2A

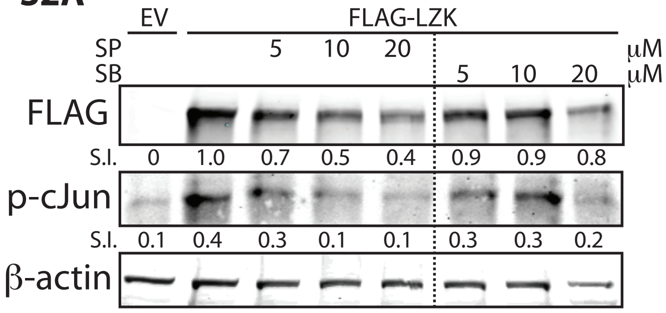

EV
FLAG-LZK
SP
5
10
20
μM
SB
5
10
20
μM
FLAG
S.I.
0
1.0
0.7
0.5
0.4
0.9
0.9
0.8
p-cJun
S.I.
0.1
0.4
0.3
0.1
0.1
0.3
0.3
0.2
β-actin

S2B

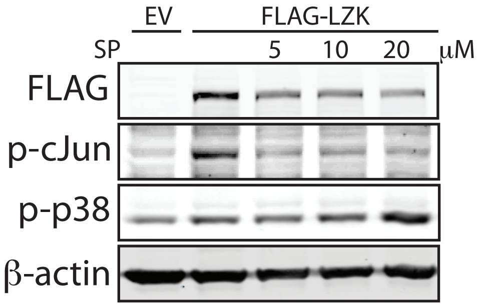

EV
FLAG-LZK
SP
5
10
20
μM
FLAG
p-cJun
p-p38
β-actin
